# Methods used for estimating sleep in dairy cattle

**DOI:** 10.3168/jdsc.2023-0474

**Published:** 2023-12-09

**Authors:** Kathryn L. Proudfoot, Emma Ternman

**Affiliations:** 1 Atlantic Veterinary College, University of Prince Edward Island, Charlottetown, C1A4P3 PEI; 2 Faculty of Biosciences and Aquaculture, Nord University, NO-7729 Steinkjer, Norway

## Abstract

Sleep serves several essential functions in all mammals including dairy cattle. Researchers are beginning to estimate sleep in dairy cattle using a combination of physiological measurements (e.g., polysomnography) as well as changes in behavior (e.g., different resting postures). Sleep may provide unique insight into how cows and calves respond to, and cope with, their environments, as a complement to other common measurements such as lying time. Although each of the methods to assess sleep in cattle has its advantages, there remain several challenges with each approach. The objective of this narrative mini-review is to describe current methods for estimating sleep in dairy cattle, including some of the advantages and limitations with each method. We will start with describing the research to date on adult cows, followed by preweaning dairy calves. We end the review with recommendations for researchers interested in assessing sleep in dairy cattle and ideas for future areas of research.

## Highlights

• Researchers are interested in including sleep as a welfare indicator in cattle.
• Sleep has been assessed using both physiology and behavior.
• Estimating sleep in adult cattle is challenging due to interference from rumination.
• Measuring sleep in calves is less challenging and may be estimated using behavior.

## Graphical Abstract

**Figure:**
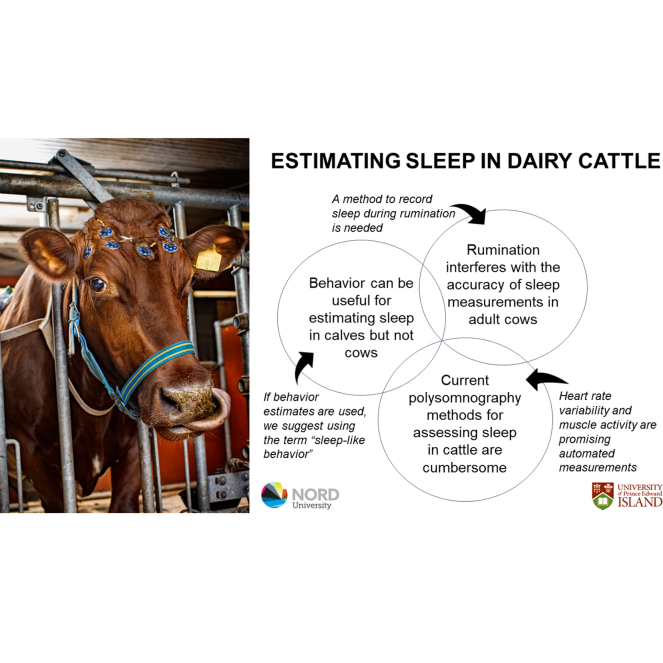
**Summary** Sleep is essential for all mammals, including dairy cattle, yet can be difficult to measure. Here, we describe the current methods used to estimate sleep in dairy cows and calves, including the limitations of each approach. We end with recommendations for researchers interested in cattle sleep.

Sleep in dairy cattle is an emerging area of scholarship for researchers working within the fields of animal science, animal welfare, and ethology. Estimations of sleep offer unique insight into how cows respond to, and cope with, their environments, as a complement to other common measurements such as lying time. Lying time is helpful to determine the quantity of physical rest a cow may receive, yet sleep can indicate the quality of that rest, and more specifically rest in the mental domain. Although underexplored, sleep can be important for the welfare of cattle, as it may serve as a mediator between stressors in a cow's environment and her health and affective states, as these links have been made in other species (discussed by Langford and Cockram, 2010). However, estimating sleep in cattle is not straightforward. The objective of this narrative mini-review is to describe current methods for estimating sleep in dairy cattle, including some of their advantages and challenges.

For all animals, sleep is considered essential for life (Keene and Duboue, 2018). In mammals, sleep is defined as a quickly reversible, calm state with an elevated stimulus threshold and species-specific posture (Zepelin et al., 2005). Dairy cattle, like other mammals, undergo different vigilance states throughout the day, including sleep and wakefulness, that can be distinguished by brain wave patterns (reviewed by Staunton, 2005). Early research on sleep in cattle defined 4 vigilance states: (1) alert wakefulness characterized by desynchronized high-frequency brain waves and obvious muscle activity, (2) an intermediate sleep state referred to as drowsing characterized by a combination of low- and high-frequency brain waves and decreased muscle activity, (3) non-rapid eye movement (**NREM**) sleep characterized by synchronized low-frequency brain waves and low muscle activity, and (4) rapid eye movement (**REM**) sleep characterized by desynchronized brain waves in addition to the absence of muscle tone and bursts of eye movements (Ruckebusch, 1972). Beginning with measurements of brain, muscle, and eye activity, researchers have identified methods to estimate sleep in dairy cattle, including distinct differences between preruminant calves and adult cows.

For many years, researchers questioned if ruminants engaged in sleep at all (e.g., Balch, 1955). However, in 1972, Ruckebusch used intracranial electroencephalography (**EEG**) electrodes implanted into the head, and electromyography (**EMG**) electrodes implanted into the musculature, to prove that cattle exhibited an EEG pattern resembling sleep in other species, including the identification of drowsing, NREM, and REM sleep states. Ruckebusch went on to map cattle sleep states under different conditions, including as pre- and postparturition, amid different feeding regimens (Ruckebusch, 1975a), and during sleep deprivation (Ruckebusch, 1974), providing much of the foundational research on sleep in dairy cows (Table 1).

**Table 1:** A summary of research studies assessing electrophysiological sleep in adult dairy cattle

Reference	Method	Main finding
Ruckebusch, 1972	Implanted electrodes^1^	Both REM^2^ and NREM^2^ sleep phases are present in adult cattle.
Ruckebusch, 1974, 1975a,b	Implanted electrodes^1^	Forced standing, pregnancy status, and type of housing affect sleep distribution.
Ternman et al., 2012	Surface electrodes^1^	Vigilance states can be distinguished using surface electrodes in adult cows.
Ternman et al., 2018, 2019	Surface electrodes^1^	Sleep recordings over 3 d vary between cows but not systematically across days. REM sleep duration changes with stage of lactation.
Kull et al., 2019; Proudfoot et al., 2021	Surface electrodes^1^	Sleep and lying deprivation reduce milk production and increase inflammatory markers.
Hunter et al., 2021a,b	Surface electrodes^1^ and heart rate variables	Heart rate and heart rate variability change based on vigilance state, and combined with EMG, can estimate sleep with high accuracy.

1 Surface and implanted electrodes include electroencephalography, electromyography (EMG), and electrooculography.

2 REM = rapid eye movement; NREM = non-rapid eye movement.

Although implanted electrodes accurately distinguished between sleep and wake states in cattle, there were several challenges with this method. A main concern is its invasiveness; the implants themselves were likely painful, which could have interfered with a cow engaging in their normal sleep patterns. An additional disadvantage is that the cows needed to be kept in restrictive environments, such as tiestalls, to prevent the device from being removed (e.g., Ruckebusch, 1972); this restriction of movement may have also influenced a cow's ability to sleep normally. A third challenge with this method is that researchers must manually score epochs of data produced from the electrodes into different sleep and wake stages, making it time consuming and subjective.

Following this initial research, methods used to assess sleep in cattle have become less invasive. For example, surface electrodes for EEG, EMG, and electrooculography (**EOG**), collectively referred to as polysomnography, have been used with good results in our research groups to record sleep and awake states in adult dairy cows (e.g., Ternman et al., 2012; Kull et al., 2019). To use these electrodes, researchers first shave areas of the cow to allow the electrode to have direct contact with the skin and sufficient conduction, then superglue the electrodes in place so that they are not likely to be removed while recording (see Ternman et al., 2012, for more details). Like the implanted electrodes, this method distinguished between states of wakefulness, drowsing, NREM, and REM sleep states (Ternman et al., 2012). Using these electrodes, Ternman et al. (2019) found that dairy cows display a polyphasic sleep pattern, with short but multiple sleep bouts throughout a 24-h period. Sleep duration and distribution, however, seems to vary between individuals, and can be affected by stage of lactation and time of day (Ternman et al., 2018, 2019).

Although surface electrodes are less invasive than implants, they still come with challenges. A main problem with using any kind of electrode to estimate sleep in ruminants is rumination; the masticatory muscles cause artifacts in the data during chewing, making it impossible to distinguish between vigilance states while the cow is ruminating, despite the ability to record rumination behavior with high accuracy (Ternman et al., 2019). This method also requires that the animal be constantly monitored to ensure that the data are accurate, and for the data to be manually scored. Cows can detach these electrodes, so researchers must keep them in more restrictive environments (e.g., individual pens; Kull et al., 2019) so that the electrodes stay in their correct placement. As social isolation is a known stressor for cows (Rushen et al., 1999), and changes in management and handling likely affect a cow's daily rhythm (DeVries and Keyserlingk, 2005), the use of surface electrodes may not allow us to make conclusions about the natural sleep patterns of cows. That said, Ternman et al. (2018) recorded sleep states for 3 consecutive days to determine if surface electrodes affected sleep patterns in cattle and discovered that sleep varied more between individuals than between days, indicating the importance of using the individuals as their own controls in cattle sleep studies.

To avoid some of the challenges of assessing sleep using implanted or surface electrodes, researchers have also explored methods to estimate sleep in cattle by recording their behavior (Table 2). This method is not new, as the definition of sleep includes reference to an animal's “species-specific posture.” Indeed, behavioral estimates are often used when studying sleep in wild herbivores such as giraffes (Tobler and Schwierin, 1996). The most common posture for sleep in large herbivores is lying down with the neck relaxed and head resting on the flank, although some herbivores also engage in sleep while standing (Zepelin et al., 2005). Cows lie down 8 to 12 h per day and are highly motivated to do so (Munksgaard et al., 2005); it is therefore likely that most of their sleep would occur lying down.

**Table 2:** A summary of research studies assessing behavioral sleep in adult dairy cattle

Reference	Method	Main finding
Norring et al., 2012	Behavior	Latency to lie down with the neck relaxed was shorter for high- compared with low-yielding cows.
Ternman et al., 2014	Behavior and surface electrodes^1^	Posture overestimates sleep in dairy cows.
Klefot et al., 2016	Behavior and triaxial accelerometer	An accelerometer-based neck sensor can moderately predict sleep-like behavior.
Fukasawa et al., 2019	Behavior and triaxial accelerometer	Season, parity, and milk components are associated with sleep-like behavior in cows.
Hunter et al., 2021c	Behavior and surface electrodes^1^	Lying posture is a poor predictor of sleep.

1 Surface electrodes include electroencephalography, electromyography, and electrooculography.

Unfortunately, posture has not been shown to be an accurate measurement of sleep in dairy cattle. For example, Ternman et al. (2014) compared different postures collected from live observations of cows with sleep states estimated using surface electrodes as a gold standard (EEG, EMG, and EOG). Behaviors included “lying with head lifted and still” as a potential predictor of NREM sleep, “lying with head resting” for REM sleep, and “lying with head lifted and moving” for wakefulness. In general, these postures poorly predict sleep states. “Lying with head lifted and still” showed high sensitivity (81%) but low specificity (6%) in predicting NREM sleep; this overestimation of NREM was likely due to cows spending a considerable amount of time drowsing in this posture. In contrast, “lying with head resting” had high sensitivity (70%) and moderate specificity (41%) for predicting REM sleep. These findings were later confirmed by Hunter et al. (2021c) for cows kept both indoors and on pasture. The moderate agreement between the REM sleep and cows lying with their head not supported by their neck may be promising as a proxy for total sleep time as the proportion of REM and NREM of total sleep time is rather stable under normal sleep conditions. However, REM sleep can be interrupted without notably affecting NREM and vice versa (Beersma et al., 1990), indicating the need for methods to accurately register both types of sleep states.

Even if behavior was a good estimate of sleep in cattle, this method still requires many hours of recording behaviors either through live observation or video. To avoid this problem, some efforts have been made to develop technology that automatically records behaviors and postures. For example, Klefot et al. (2016) used triaxial accelerometers attached to the halter of dairy cows to predict the head position of cows while sleeping. However, authors used behavior as the gold standard for sleep, rather than a more accurate predictor of sleep such as polysomnography. Thus, researchers assessing new technology for estimating sleep are encouraged to use more accurate assessments of sleep as their gold standard.

In addition to polysomnography, researchers have also attempted to assess sleep using other physiological indicators. For example, Hunter et al. (2021a) used heart rate (**HR**) and heart rate variability (**HRV**) collected from HR monitors on cows to estimate sleep states from EEG, EMG, and EOG surface electrodes. Unlike other studies using dairy cows, these authors classified NREM sleep into 3 stages: light N1, N2, and deep slow-wave N3 sleep. The researchers found that both HR and HRV changed depending on sleep state for cows kept both indoors and on pasture. For example, HR slowed down between wake and NREM sleep and was the lowest during REM sleep. Heart rate variability, assessed using the root mean square of successive differences, was highest during REM, indicating more variability between successive heart beats during this stage compared with both wakefulness and NREM.

In a follow-up study, Hunter et al. (2021b) used a combination of neck muscle activity (EMG) and heart rate measurements (HR and HRV) to predict sleep states in adult cows. Tools to measure muscle activity and HR still require some attachment to the animal but may be more practical than using cumbersome polysomnography electrodes; for example, the EMG and HR/HRV data can be automatically generated from the sensors and do not require manual interpretation of the data. Using machine learning models (neural networks and random forest algorithms), Hunter et al. (2021b) found that a combination of data generated from the HR monitor and EMG could accurately predict 82.3% of sleep stages recorded manually from polysomnography. The most accurate predictions were for wakefulness and REM sleep; the models could predict wakefulness with a 94% chance of a correct score and REM sleep with 92% chance of a correct score. Of the 15 different features of the HR and EMG data included in the machine learning models, the most informative were those from the neck muscle (neck root mean square, variance, and SD), likely because muscle tone is low during NREM sleep and completely relaxed during REM sleep (Ruckebusch, 1972).

Like other methods, the use of HR and muscle activity as indicators of sleep in cattle still have their challenges. A main concern, like the use of surface electrodes, is that these tools cannot be used accurately when the cow ruminates. Indeed, Hunter et al. (2021b) excluded any epochs of data that contained rumination or when the cow was standing; thus, their model can only predict sleep when the cow is lying down and not ruminating.

Although rumination seems to be a main problem in assessing sleep in adult cattle, this is less of a problem for preruminant dairy calves (Table 3). In one of the first studies of sleep in dairy calves, Hänninen et al. (2008a) investigated whether calf posture could predict sleep states estimated using polysomnography and discovered that, unlike cows, some postures were fairly good at predicting sleep states. For example, a calf “resting head lifted up still” predicted 55% of NREM sleep epochs, and “resting with neck relaxed” predicted 61% of REM sleep epochs. Most promising, the combination of these 2 behaviors achieved 82% specificity and 78% sensitivity at predicting total sleep time in calves (NREM and REM). Using these data, Hänninen et al. (2008a) found that calves, like cows, engage in several short periods of sleep (e.g., approximately 50 bouts of 5 min over a 20-h period) rather than 1 or 2 longer bouts. Posture is likely a better predictor of sleep in calves because they do not engage in the intermediate stage of drowsing like adult cows.

**Table 3:** A summary of research studies assessing behavioral and electrophysiological sleep in dairy calves

Reference	Method	Main finding
Hänninen et al., 2003	Behavior	Group housing and body temperature can affect REM-type^1^ sleep-like behavior.
Hänninen et al., 2008a	Surface electrodes^2^ and behavior	Posture is moderately predictive of sleep.
Hänninen et al., 2008b	Behavior	Milk feeding method and separation from the dam affects sleep-like behavior.
Hokkanen et al., 2011	Triaxial accelerometer and behavior	An accelerometer-based neck sensor can predict sleep-like behavior.
Adcock et al., 2023	Behavior	Disbudding can increase sleep-like behavior.
Fukasawa, 2023	Behavior	Sleep-like posture changes as calves age.

1 REM = rapid eye movement.

2 Surface electrodes include electroencephalogram (EEG), electromyography (EMG), and electrooculography (EOG).

However, like adult cows, there remain challenges with using surface electrodes to estimate sleep in calves. For example, Hänninen et al. (2008a) opted to sedate the calves to attach the electrodes to minimize distress during handling, which adds an extra step to the process and might alter subsequent sleep architecture (Luo et al., 2020). This method may also limit the housing of calves to more restrictive environments to protect the electrode placement. However, the finding that posture may be a good predictor of sleep states has allowed for more automated assessments of sleep in calves. For example, Hokkanen et al. (2011) created a neck-based, wireless triaxial accelerometer that could distinguish 82% of the occurrence of sleep measured from calf postures recorded from live observation. The accelerometer, and subsequent model generated from its data, could also distinguish 66% of the time that calves were in a posture indicating NREM, and 70% of the time the calves were in a posture indicating REM sleep. Although promising, this type of device still has its challenges, as it overestimated postures indicating NREM and underestimated postures indicating REM. Moreover, these postures were also not perfect predictors of NREM and REM (Hänninen et al., 2008a), making the device likely even less accurate as a tool to assess different sleep states.

We end this review with recommendations for researchers who plan to estimate sleep in dairy cows and calves. For adult cattle, polysomnography using surface electrodes remains the most practical and accurate method for assessing sleep. However, researchers should be cautious of some of the limitations of this approach and challenges in interpreting this data caused by rumination. Researchers who choose to use behavior as a proxy for sleep in adult cows must recognize that these are generally poor predictors of sleep; we recommend that researchers use the term “sleep-like behavior” rather than “sleep” to be clear that these behaviors may not be indicating true changes in brain activity associated with sleep states.

Behavioral measurements are a better proxy for sleep in dairy calves than adult cows. If using behavior to estimate sleep in calves, we recommend continuous data collection, as to our knowledge there has not yet been any scan sampling method validated for sleep-like behaviors in calves. It is also unlikely that scan sampling will adequately capture calf sleep-like behaviors as they occur in frequent short bouts throughout the day (Hänninen et al., 2008a). Researchers should also be aware that behavior was a better predictor of total sleep time in calves, but less accurate at distinguishing between sleep states (NREM and REM). Thus, if researchers are interested in estimating NREM or REM states separately, polysomnography remains the best approach.

Next, we encourage further development of accurate, automated sleep recording methods that can be used for cows and calves in group housing systems and for long-term studies on sleep. For example, assessments of HR and muscle activity seem like a promising next step for estimating sleep in adult cows in groups (Hunter et al., 2021a,b); however, these tools have not been able to resolve problems with detecting sleep during rumination, requiring additional time-consuming behavioral analysis to detect rumination and lying behavior. Thus, more research is encouraged to make these tools more time and cost efficient before they can use used on large numbers of animals over a long period. For calves, these tools may also be useful to help distinguish between sleep states, as this is currently a limitation of recording sleep-like behaviors; thus, researchers are encouraged to test similar tools in young dairy calves and growing heifers. Other physiological changes, such as respiration and body temperature, may also be useful for estimating sleep in cattle as they are used in other species (Douglas et al., 1982; Gilbert et al., 2004), especially if used in combination with other measurements. We encourage researchers to use polysomnography, although cumbersome, to help validate the use of these novel tools as predictors of sleep.

Adult cows display rumination and drowsing in addition to sleep and awake, 2 states that are not present in calves' behavioral repertoire. There is no clear definition of drowsing, nor a description of function of the state, but according to Ruckebusch (1972), it appears frequently during rumination. The state may be viewed as light sleep as features indicative of human sleep stage 1 have been described during drowsing in cows (Ternman et al., 2012). Moreover, Hunter et al. (2021a,b,c) classified drowsing as light N1 sleep. As it is not possible to accurately record vigilance state during rumination using the noninvasive electrodes, there are several hours per day where information about a cow's vigilance state is not accounted for. There have been attempts to remove the artifacts caused by the masticatory movements by applying different digital filters (Pastell et al., 2012). The results look promising but further work on validating the accuracy of the filtered data is needed before the method can be fully applied.

The objective of this mini-review was to describe current methods for estimating sleep in dairy cattle, including some of the advantages and challenges with each method. Early research with adult cattle used implanted electrodes to assess sleep; newer and less invasive methods include the use of surface electrodes for measurements of polysomnography (EEG, EMG, and EOG) in cows and calves. Although polysomnography remains the most accurate method to assess sleep in cattle, it is cumbersome to use, requires animals to be housed in restrictive environments, and entails substantial efforts to manually score and interpret the data. In addition, digital filters to denoise EEG data during rumination still need to be validated to accurately estimate total sleep duration in cows. Automated measurements of HRV and muscle activity are promising novel methods to predict sleep in cows, though more research is needed to refine these tools and apply them to calves. Behaviors are poor predictors of sleep for adult cows but are better at estimating sleep in young calves. Automated assessments of sleep-like behaviors using accelerometers exist for both cows and calves, yet researchers using these tools must consider their limitations. Further research is encouraged to identify novel, automated tools to assess sleep in cows and calves, as well as further develop our understanding of drowsing and its relationship to rumination in cattle.
